# Comprehensive Behavioural Analysis of Long Evans and Sprague-Dawley Rats Reveals Differential Effects of Housing Conditions on Tests Relevant to Neuropsychiatric Disorders

**DOI:** 10.1371/journal.pone.0093411

**Published:** 2014-03-26

**Authors:** Karly M. Turner, Thomas H. J. Burne

**Affiliations:** 1 Queensland Brain Institute, The University of Queensland, St Lucia, Australia; 2 Queensland Centre for Mental Health Research, The Park Centre for Mental Health, Wacol, Australia; Radboud University, Netherlands

## Abstract

Genetic (G) and environmental (E) manipulations are known to alter behavioural outcomes in rodents, however many animal models of neuropsychiatric disorders only use a restricted selection of strain and housing conditions. The aim of this study was to examine GxE interactions comparing two outbred rat strains, which were housed in either standard or enriched cages. The strains selected were the albino Sprague-Dawley rat, commonly used for animal models, and the other was the pigmented Long Evans rat, which is frequently used in cognitive studies. Rats were assessed using a comprehensive behavioural test battery and included well-established tests frequently employed to examine animal models of neuropsychiatric diseases, measuring aspects of anxiety, exploration, sensorimotor gating and cognition. Selective strain and housing effects were observed on a number of tests. These included increased locomotion and reduced pre-pulse inhibition in Long Evans rats compared to Sprague Dawley rats; and rats housed in enriched cages had reduced anxiety-like behaviour compared to standard housed rats. Long Evans rats required fewer sessions than Sprague Dawley rats to learn operant tasks, including a signal detection task and reversal learning. Furthermore, Long Evans rats housed in enriched cages acquired simple operant tasks faster than standard housed Long Evans rats. Cognitive phenotypes in animal models of neuropsychiatric disorders would benefit from using strain and housing conditions where there is greater potential for both enhancement and deficits in performance.

## Introduction

Complex neuropsychiatric disorders, such as schizophrenia and autism, are affected by multiple genetic and environmental risk factors, possibly through the interaction between a vulnerable genotype (G) and an adverse environmental (E) ‘hit’ [1]–[3]. For example, cannabis use and having the catechol-o-methyl transferase (COMT) valine allele are separately implicated as schizophrenia risk factors [4], [5] and the functional polymorphism in the COMT gene has been found to alter the effects of cannabis [6]–[8]. However, schizophrenia is associated with hundreds of genes of small effect rather than a few genes of large effect [9]. Genetic models of relevance to schizophrenia have investigated the functions of individual candidate genes [10]–[15]. While these models provide important information about the gene of interest, they cannot encapsulate the polygenic nature of this disorder. On the other hand, rodent strains were bred to produce consistent strain-dependent phenotypes that differ on a range of behavioural and physiological measures [16]–[20]. Comparing strains may provide an avenue to investigate how polygenetic vulnerability interacts with environmental conditions to produce deficits relevant to neuropsychiatric disorders [21]. For example, a comparison of Sprague-Dawley (SD), Lewis and Fischer 344 rat strains demonstrated that F344 rats, which are more responsive to stress [16] had the greatest vulnerability to a neonatal ventral hippocampal lesion [22] which is used as a neurodevelopmental animal model of schizophrenia.

Both genetic and environmental risk factors have been investigated using animal models of neuropsychiatric disorders, however most of these manipulations have only been tested under standard housing conditions. Standard housing conditions vary between facilities and over time, however generally this is referring to a rather barren cage containing nesting material and minimal in dimensions. Environmental enrichment in rodents incorporates greater sensory, cognitive and motor stimulation [23] and may be considered therapeutic, or conversely standard housing may be seen as impoverished [24], [25]. Whether effects of enrichment are positive or negative may also depend on the animal model or disorder being investigated. Either way, enriching the housing conditions of laboratory rodents has been found to alter behaviour, stress hormone levels, neurogenesis, dendrite structure, and gene expression (see review [26]. We need to consider how standard housing conditions are impacting on brain development, especially with increasing research focussed on GxE interactions [27]. Despite evidence that environmental enrichment has reversed or retarded deficits in animal models of neurological disorders, few studies have assessed these effects in rodent models of schizophrenia [28].

The validity of both genetic and environmental animal models has been assessed using tests that are relevant to the disorder being modelled. With many neuropsychiatric disorders diagnosed based on cognitive and behavioural symptoms, assessment of animal models also relies on measuring relevant behavioural characteristics [29]. Using tests employed in behavioural screens, strain and housing conditions have been shown to affect multiple behavioural domains including locomotion, anxiety, pre-pulse inhibition and acquisition of operant tasks. However, few papers have directly evaluated strain and housing manipulations across a broad screen of behavioural tests in the same study, making comparisons between tasks difficult and often conflicting [26]. Therefore, the aim of this experiment was to behaviourally phenotype pigmented Long Evans (LE), which are commonly used in cognitive experiments, and compare them to albino SD rats, which are frequently used to model neuropsychiatric disorders after they are reared in either standard or enriched housing conditions. Tests used in the behavioural test battery were selected to measure a broad range of behaviours that are frequently assessed in animal models of neuropsychiatric disorders. These tests highlight the behavioural alterations between strains and housing conditions and suggest animal models should consider using different strains for different purposes.

## Materials and Methods

### Animals and housing

Nine-week old male SD (Asmu:SD) and LE (Asmu:LE) rats were both obtained at weaning (3 weeks of age) from Monash Animal Services (Melbourne, Australia) to ensure transport and housing prior to weaning were equivalent. They were then housed in a room maintained at 21 ± 2°C and 60% humidity and on a 12-h light/dark cycle (lights on 0600 h). Standard rat chow (Specialty Feeds, WA, Australia) and water were supplied *ad libitum*. On arrival rats were pair-housed in either standard or enriched cages (n = 8/strain/housing) with groups balanced for initial body weight and pairs matched to avoid excessive dominance. Standard housing consisted of a polypropylene cage (41×28×24 cm) with a high top wire lid, aspen chip bedding (Able Scientific, WA, Australia), nesting, and wood chew (Able Scientific, WA, Australia). The alternative enriched housing condition used a larger sized polypropylene cage (54×36×30 cm) with a high top wire lid, bedding, nesting, wood chew, an enclosed shelter (15×15×12 cm) and running wheel (20.3 cm diameter, Super Pet Run-Around Wheel, IL, USA). Rats were weighed weekly and all testing was conducted during the light phase. Bicycle computers were used to record running wheel rotations (Bontrager Trip 2, Trek Bicycle Corporation, WI, USA). Rats were observed to play and jump on the wheels as well as running as a pair simultaneously as juveniles, which interfered with the accuracy of running distance. However, the values recorded confirmed the wheels were used particularly during the dark phase (data not presented). At the end of the experiment the rats were used in a separate operant-based study to be reported elsewhere. All procedures used were performed with the approval from The University of Queensland Animal Ethics Committee, under the guidelines of the National Health and Medical Research Council of Australia.

### Behavioural test battery

The effects of strain and housing conditions were characterised using a behavioural test battery that began at 9 weeks and concluded at 12 weeks of age. This was followed by a 3-week rest period and one week of food restriction before operant training started. Rats were weighed weekly and food restriction was delayed until adulthood when free-feeding weight stabilised to avoid changes in growth. The tests selected assessed a range of domains including anxiety-related behaviour, spatial memory, exploration, locomotion and sensorimotor gating. These tests have been widely used and standard protocols were adopted [30]–[32]. Tests were conducted during the light phase on separate days in the following order to reduce the influence of test order: elevated plus maze, hole board, light-dark emergence, open field, 8-arm radial maze, Y-maze, pre-pulse inhibition (PPI) of the acoustic startle response and finally operant task acquisition. Although the elevated plus maze may be more stressful than tests conducted afterwards, it is also very sensitive to order effects and hence was conducted first. An extra rest day was included after the elevated plus maze. All behaviours were recorded using automated software or scored blind to treatment. Computerised tracking software, EthoVision ver.3.1 (Noldus, Netherlands) was used to analyse video recordings from a camera mounted above each testing arena. Ethologically relevant behaviours were scored using Observer ver.5.0 (Noldus, Netherlands). Rats were habituated to the testing room 30 minutes prior to testing and the apparatus was cleaned with 70% ethanol between each trial.

#### Elevated plus maze (EPM)

The elevated plus maze was used to measure anxiety-related behaviours, but also provided measures of exploration and locomotion [33]. The plus-shaped platform was made of opaque grey plastic, elevated on a stand (70 cm) with two arms enclosed by walls (10×47×40 cm; 1 lux) and the other two arms open (10×47 cm; 8 lux). Rats were placed in the centre of the maze facing an open arm and allowed to explore the maze for 10 minutes. Measures included percentage of time spent on the open arms (% Open  =  (time spent open arms/(time spent open arms + time spent closed arms)) ×100) and distance travelled. These results were further investigated by scoring ethologically relevant behaviours such as rearing, grooming and risk assessment behaviours were also scored. Rearing was defined as standing with fore limbs lifted; grooming was scored when the animal was licking and cleaning their body; head dip was scored when the rats head was pointed downward over the edge of the platform; scanning was scored when the rats head was pointed outward and upward while the rat was stationary; and stretch attend posture was defined as stretching the head forward without stepping forward. If behaviours were split for zone, protected describes behaviours in the closed arms and centre zone, while open describes behaviours that occurred on the open arms.

#### Hole board

The hole board test was used to measure exploration and locomotion [34]. An opaque grey arena (60×60×40 cm; 8 lux in centre) with an elevated floor containing four holes (4 cm wide, 12.5 cm from each corner) was used. The rat was placed into a corner and explored the arena for 10 minutes. Measures included the distance travelled, time spent in the centre of the arena and the duration, frequency and latency to perform head dipping, grooming and rearing behaviour.

#### Light dark emergence

Light dark emergence was used as an alternative measure of anxiety-related behaviours and exploration [35]. The dark compartment provides shelter and an increase in the time taken to leave the shelter to explore the open compartment is indicative of greater anxiety. A plastic arena (44×44×30 cm, 7 lux in open) was divided such that half the arena was open and the other half was completely enclosed with the exception of a central doorway (8×11 cm). To start the 10-minute trial the rat was positioned at the doorway and required to enter the dark section. The key measures were time to emerge from the dark chamber and the number of transitions between the light and dark sections.

#### Open field

The open field test was used to measure locomotion, however the use of different zones within the arena also provides information on anxiety-related behaviours [36]. Rats were placed in the centre of an open arena (60×60×40 cm; 5.5 lux) and tracked over 10 minutes. Measures included distance travelled, time spent in the centre and crossings into the centre.

#### 8-Arm radial maze

The 8-arm radial maze has been used for a number of protocols to assess different aspects of cognition. A simplified protocol was used in this study to investigate exploratory behaviour in a novel arena [37], [38]. The opaque grey plastic maze consisted of 8 arms (60×10×20 cm, 3 lux) joined by a central octagon (25.5 cm wide; 8 lux). The rat was placed in the centre and the order and timing of arm visits was recorded over 10 minutes. The rat should visit each arm once until all arms have been explored. Revisiting an arm prior to exploring all 8 was considered an error. The number of errors and time taken to visit all the arms were recorded, as well as distance travelled.

#### Y-maze

Three arms of the 8-arm radial maze were used for this test and the other arms were blocked off at the centre. Two trials were conducted to investigate short-term recognition memory. During the first trial the rat was placed at the end of the ‘home’ arm and given access to one other arm, referred to as the ‘familiar’ arm for 10 minutes. After an inter-trial interval of one hour the rat was placed back in the home arm and has access to the familiar arm in addition to a previously occluded arm, the ‘novel’ arm for 5 minutes. The amount of time spent in each arm and the number of transitions was recorded. The rat should spend more time exploring the novel arm if it has remembered visiting the home and familiar arms in the previous trial. Within each pair the familiar and novel arm was alternated between the left and right arms. The ends of these arms were decorated with either vertical or horizontal barred patterns to facilitate recognition.

#### Pre-Pulse Inhibition (PPI) of Acoustic Startle Response (ASR)

PPI was used as a measure of sensorimotor gating, whereby a startle reflex is reduced if a weaker pre-pulse precedes the startling pulse. Responses were recorded in startle chambers by placing rats into clear Plexiglas cylinders on platforms connected to piezoelectric transducers, which were housed in sound attenuating chambers containing speakers and controlled using specialist software (SR-Lab, San Diego Instruments). The session consisted of pseudo-randomised presentation of the different trial types. The acoustic startle response (ASR) was measured using a single 40 ms pulse at various intensities (70, 80, 90, 100, 110, 120 dB) and within-session habituation was measured as the change in startle response to a 110 dB pulse presented at the start, middle and end of the session. Pre-pulses at a three different intensities (74, 78, 86 dB) were played at a variety of intervals (8, 16, 32, 64, 128, 256 ms) prior to the startle pulse (120 dB) to assess pre-pulse inhibition. Each trial type was presented five times and the median was used for further analysis. Percentage PPI was calculated as follows: [(startle amplitude of ASR trial - startle amplitude on prepulse trial)/startle amplitude of ASR trial] ×100.

### Operant training

Training was conducted in operant chambers housed in ventilated, sound attenuating boxes (50×50×50 cm, Med Associates Inc., St. Albans, VT, USA). Rats were initially trained to collect a reward (45 mg, F0021, dustless precision pellet, Bioserv, Frenchtown, NJ, USA) from one of two receptacles equipped with head entry detectors that were located on the left and right side of the wall. Every head entry was rewarded with one pellet until 50 pellets were delivered from each receptacle or until the session ended after 20 mins. Once rats had attained >80 pellets on 2 days they were trained to nose poke a central aperture when it was illuminated to receive a reward, which was delivered to either the left or right receptacle. Finally, after learning to initiate trials by nose poking, the signal detection task was implemented. After initiating trials with a nose poke, a panel of 9 green LEDs were either illuminated (signal trial) or remained off (non signal trial). After 1 s both the left and right magazines illuminated to indicate the rat should make a choice. Depending on the visual cue presented, a head entry into one side would lead to a pellet and the other had no consequence. The pairing of a trial type (signal or non signal) and the correct magazine side (left or right) remained the same for each individual but was balanced across the group. Between trials there was a variable inter-trial interval (1, 2, 3 s) and the session concluded after either 100 trials or 30 min. The chamber was operated using MED-PC for Windows software and interfacing (Med Associates Inc., St. Albans, VT, USA).

### Statistical analysis

Results were analysed using SPSS software (ver. 20, SPSS Inc., Chicago, Illinois). A power analysis determined that a group size of n = 8 was required to detect a difference with an effect size of 0.85 with 95% power and at the criterion of *p*<0.05. Key behavioural measures were assessed for skewness and kurtosis for confirm the assumption of a normal distribution. The main effect of Strain (SD or LE) and Housing (standard or enriched) on key parameters for each test was subjected to an ANOVA or repeated measures ANOVA was applied where required. Significant interactions were then assessed using independent *t*-tests. Due to the large difference in variation, the effect of housing was assessed in each strain separately for PPI. Data is presented as mean ± standard error of the mean (SEM) and statistical significance was determined if *p*<0.05.

## Results

**Figure 1:**
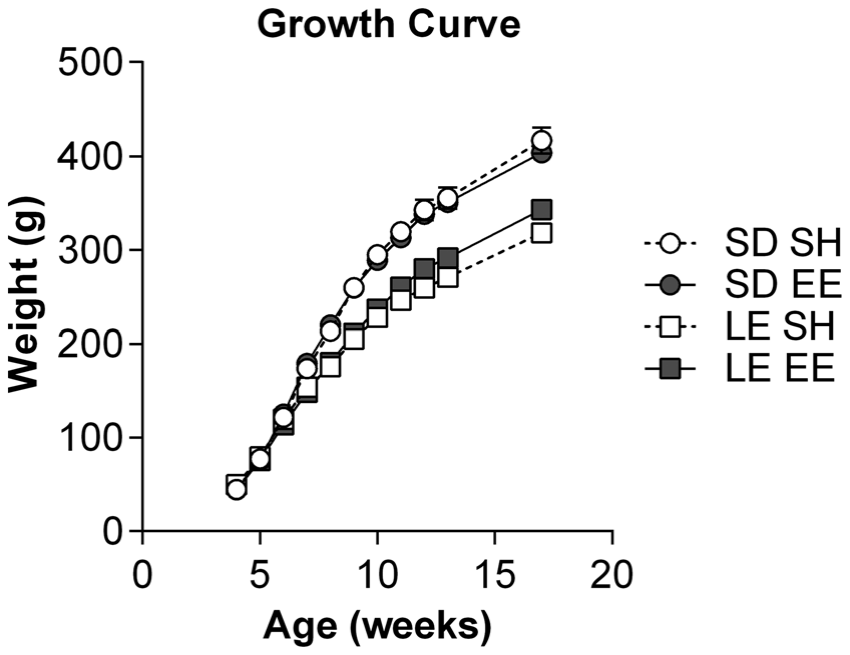
Growth Curve. The body weight of Sprague Dawley (SD, circles) and Long Evans (LE, squares) rats from 3–16 weeks of age housed in either standard (SH, open) or enriched (EE, closed) housing. Overall, there was a main effect of strain (*F*_(1,28)_ = 54.75, *p*<0.001), but not housing (*F*_(1,28)_ = 0.24, *p* = 0.625). LE rats tended to separate more towards the end of the experiment, however this was not significant even at 16 weeks (*t*_(10.85)_ = 2.18, *p* = 0.052).

The body weight of Long Evans and Sprague Dawley rats housed in either enriched or standard housing was recorded from 3–16 weeks of age. Overall, there was a main effect of strain (*F*_(1,28)_ = 54.75, *p*<0.001), but not housing (*F*_(1,28)_ = 0.24, *p* = 0.625; Fig.1). LE rats from standard and enriched housing tended to deviate more towards the end of the experiment, however this was not significant even at 16 weeks (*t*_(10.85)_ = 2.18, *p* = 0.052).

### Behavioural test battery

Due to the number of behavioural parameters reported, the main measures from the behavioural test battery are presented in Table 1, with ethological measures presented in Table 2.

**Table 1:** Behavioural Test Battery. Key behavioural parameters from each test in the behaviour screen for Sprague Dawley and Long Evans rats housed in standard or enriched conditions.

	Sprague Dawley		Long Evans		Strain (*F*_(1,31)_)	Housing (*F*_(1,31)_)	Interaction (*F*_(1,31)_)
Test and measure	Standard (n = 8)	Enriched (n = 8)	Standard (n = 8)	Enriched (n = 8)			
*Elevated Plus Maze*							
Distance (cm)	4057.2 ± 227.4	3863.5 ± 452.7	5555.0 ± 239.3	4494.1 ± 439.5	8.93**	3.10	1.48
Duration in Centre (s)	153.5 ± 15.8	112.2 ± 10.4	122.3 ± 13.9	125.2 ± 12.8	0.46	2.07	2.73
% Duration Open Arm	31.4 ± 5.6	10.3 ± 3.6	42.6 ± 4.4	26.6 ± 4.0	9.45**	17.24**	0.31
*Hole board*							
Distance (cm)	3929.7 ± 186.2	3538.7 ± 261.9	4740.5 ± 150.8	4449.1 ± 268.1	14.97**	2.35	0.05
Time in centre (%)	2.1 ± 0.8	3.1 ± 0.9	5.7 ± 1.0	4.9 ± 1.3	7.21*	0.09	0.88
Latency Head Dip	60.5 ± 14.7	12.7 ± 4.1	29.2 ± 5.8	24.0 ± 5.7	1.35	9.48**	6.10*
*Light Dark Test*							
Distance (cm)	1437.5 ± 94.7	1303.4 ± 185.2	2477.1 ± 251.7	2230.6 ± 236.5	23.79**	0.89	0.08
% Duration in Open	54.4 ± 2.7	45.5 ± 6.2	62.7 ± 5.2	63.4 ± 5.7	6.54*	0.64	0.88
Latency to Open (s)	20.3 ± 4.1	7.6 ± 1.7	12.7 ± 1.8	8.8 ± 1.5	1.61	10.93**	3.02
*Open Field*							
Distance (cm)	3485.0 ± 250.7	2832.4 ± 277.6	4189.0 ± 381.3	3169.9 ± 148.2	3.53	9.09**	0.44
Time in centre (%)	3.7 ± 1.1	3.6 ± 1.0	6.7 ± 1.7	4.9 ± 0.4	3.62	0.72	0.63
Entries to centre	11.0 ± 2.7	8.8 ± 1.7	17.9 ± 3.4	12.9 ± 1.2	5.17*	2.25	0.32
*Y-Maze*							
Distance Day 2 (cm)	2919.3 ± 90.2	2968.9 ± 174.3	3183.5 ± 210.5	2706.4 ± 125.7	0.00	1.85	2.81
Novel vs Fam Dur	63.1 ± 5.1	71.6 ± 3.1	59.2 ± 6.7	63.5 ± 6.2	1.22	1.37	0.14
Novel arm entries	6.4 ± 1.0	6.3 ± 0.3	7.9 ± 1.0	5.4 ± 0.6	0.16	2.75	2.25
*Radial Arm Maze*							
Distance (cm)	5770.4 ± 706.5	6424.6 ± 424.0	7709.3 ± 411.9	7692.0 ± 729.1	7.63*	0.30	0.33
Time to complete	226.7 ± 17.4	188.6 ± 19.0	137.0 ± 10.5	139.9 ± 8.5	23.22**	1.50	2.03
Entries to complete	12.0 ± 0.7	12.0 ± 0.8	9.6 ± 0.5	9.6 ± 0.5	14.75**	0.00	0.00

Data are presented as mean ± SEM and statistical results for strain comparison. Main effect of Strain and Housing was assessed by ANOVA and independent-samples *t*-tests were then performed if a significant Strain*Housing interaction was detected; *p<0.05, **p<0.01.

**Table 2:** Ethological measures. Behavioural observations from EPM and Hole board tests showing the mean and SEM for each group.

	Sprague Dawley		Long Evans		Strain (*F*_(1,31)_)	Housing *F(*_(1,31)_)	Interaction (*F*_(1,31)_)
Test and measure	Standard (n = 8)	Enriched (n = 8)	Standard (n = 8)	Enriched (n = 8)			
*Elevated Plus Maze*							
Latency to Protected Groom	338.5 ± 68.3	233.7 ± 53.1	234.4 ± 61.0	116.2 ± 24.3	4.31*	4.37*	0.16
Duration Groom (s)	8.0 ± 2.4	30.5 ± 12.4	24.8 ± 7.4	113.6 ± 24.3	12.45**	15.41**	5.47*
Freq. Groom	3.3 ± 0.9	4.9 ± 1.1	5.1 ± 1.3	6.5 ± 1.0	2.69	1.98	0.01
Duration Protected Scanning (s)	61.8 ± 7.7	67.7 ± 9.9	27.5 ± 4.4	35.4 ± 3.2	23.78**	1.03	0.02
Freq. Open Head Dip	11.3 ± 3.0	3.0 ± 1.0	22.5 ± 2.9	16.9 ± 2.4	26.34**	8.03**	0.29
Freq. PSA	4.3 ± 0.8	5.1 ± 0.8	2.0 ± 0.9	5.6 ± 1.0	1.00	6.58*	2.46
Latency PSA (s)	162.5 ± 35.2	69.4 ± 13.8	164.7 ± 41.9	128.0 ± 26.1	1.04	4.74*	0.90
*Hole board*							
Latency to Groom (s)	229. ± 29.7	231.8 ± 57.0	204.9 ± 52.2	165.5 ± 56.6	0.82	0.14	0.18
Duration Groom (s)	2.9 ± 0.6	7.0 ± 3.3	4.6 ± 0.8	7.8 ± 2.1	0.44	3.43	0.05
Freq. Groom	3.9 ± 0.8	3.8 ± 1.0	5.0 ± 1.1	5.8 ± 0.8	2.76	0.11	0.22
Freq. Head Dip	23.0 ± 2.1	24.8 ± 3.7	34.4 ± 3.9	30.5 ± 2.8	7.15*	0.11	0.77
Freq. Rear	42.1 ± 4.0	41.9 ± 7.3	56.9 ± 3.5	54.6 ± 7.6	5.45*	0.05	0.03

Data are presented as mean ± SEM and statistical results for strain comparison. Main effect of Strain and Housing was assessed by ANOVA and independent-samples *t*-tests were then performed where a significant Strain*Housing interaction was detected. *p<0.05, **p<0.01. PSA = Protected Stretch Attend posture.

#### Elevated plus maze

There was a significant effect of both Strain (*F*_1,31_ = 9.45, *p* = .005) and Housing (*F*_(1,31)_ = 17.24, *p*<0.001) but no interaction (*F*_(1,31)_ = 0.31; *p* = .582) on the percentage of time spent on the open arm of the EPM. LE rats spent significantly longer on the open arm than SD rats, and standard housed rats spent significantly longer on the open arms than those from enriched cages. There was no significant difference between Strains, however SD rats from enriched cages spent less time than those from standard housing in the centre of the maze (*t*_(14)_ = −2.19, *p* = .046). The total distance travelled on the EPM differed based on Strain, such that LE rats had greater locomotion than SD rats (*F*_(1,31)_ = 8.93, *p* = .006). Within the ethological behaviours, it was found that enriched rats performed protected stretch-attend risk assessment behaviour earlier (*F*_(1,31)_ = 4.74, *p* = .039) and more frequently (*F*_(1,31)_ = 6.58, *p* = .016) than standard housed rats. There was no significant effect of Strain detected for these measures, however when analysed separately frequency of protected stretch-attend behaviour was only greater in enriched LE compared to those in standard housing (*t*_(14)_ = 2.66, *p* = .019). An analysis of grooming behaviour revealed main effects of Strain and Housing on latency (*F*_(1,31)_ = 4.31, *p* = .048; *F*_(1,31)_ = 4.37, *p* = .046) and duration (*F*_(1,31)_ = 12.45, *p* = .001; *F*_(1,31)_ = 15.4, *p* = .001) with a significant Strain*Housing interaction (*F*_(1,31)_ = 5.47, *p* = .027) but no significant effect on frequency of grooming. These results indicated LE rats groom earlier and for longer than SD rats, and that rats from enriched housing groomed earlier and for longer than those from standard housing. A post-hoc *t*-test revealed LE rats housed in enrichment groomed for significantly longer than those from standard housing (*t*_(14)_ = 3.50, *p* = .004).

#### Hole board

A main effect of Strain was found for distance travelled on the hole board with LE rats travelling significantly further than SD rats (*F*_(1,31)_ = 14.97, *p* = .001) and spending more time in the centre zone (*F*_(1,31)_ = 7.21, *p* = .012). The latency to head dip differed by Housing condition (*F*_(1,31)_ = 9.48, *p* = .005) and there was a significant interaction of Strain*Housing (*F*_(1,31)_ = 6.10, *p* = .020). SD rats reared in standard housing took significantly longer to head dip on the hole board compared to those housed in enriched cages (*t*_(14)_ = −3.14, *p* = .007). Frequency of head dipping (*F*_(1,31)_ = 7.15, *p* = .012) and rearing (*F*_(1,31)_ = 5.45, *p* = .027) was significantly greater in LE rats compared to SD rats, but there was no main effect of Housing.

#### Light dark emergence

The key measure of the light dark test was the latency to enter the open section and there was a significant effect of Housing in SD rats (*t*_(14)_ = −2.85, *p* = .013), without a main effect of Strain (*F*_(1,31)_ = 1.61, *p* = .215). By contrast there was a main effect of Strain (*F*_(1,31)_ = 6.54, *p* = .016) but not Housing (*F*_(1,31)_ = 0.64, *p* = .432) on the percentage of time spent in the open. An effect of Strain (*F*_(1,31)_ = 23.79, *p*<0.001) was again detected for distance travelled, indicating LE rats had increased locomotion compared to SD rats.

#### Open field

While distance travelled was found to differ between strains on a number of other tests, no significant effect of Strain (*F*_(1,31)_ = 3.53, *p* = .071) was found on the open field test. However, there was a main effect of Housing (*F*_(1,31)_ = 9.09, *p* = .005), in which enriched rats moved less than standard housed LE rats. While the total distance travelled after 10 minutes differed between strains, these groups did not differ after the 1^st^ minute time bin, indicating altered habituation. There was a significant effect of Strain (*F*_(1,31)_ = 5.17, *p* = .031) on number of crossings into the centre, but no significant difference between groups on the percentage of time spent in the centre.

#### 8-Arm radial maze

Each rat visited all 8 arms of the maze. There was a main effect of Strain, but not Housing, on the time to enter all 8 arms (*F*_(1,30)_ = 23.22, *p*<0.001), the total number of arms entered to visit all 8 arms (*F*_(1,30)_ = 14.75, *p*<0.001) and the distance travelled (*F*_(1,30)_ = 7.63, *p* = .010). LE rats completed the 8-arm radial arm maze faster and with fewer errors than SD rats.

#### Y-maze

The percentage of time spent in the novel arm was greater than the familiar arm in each group, indicating rats were able to recognise the previously visited arm after a one-hour inter-trial interval. There was no significant effect of Strain or Housing on the percentage of time spent in the novel vs. familiar arm, the number of novel arm entries or the distance travelled.

#### Pre-Pulse Inhibition (PPI) of Acoustic Startle Response

**Figure 2:**
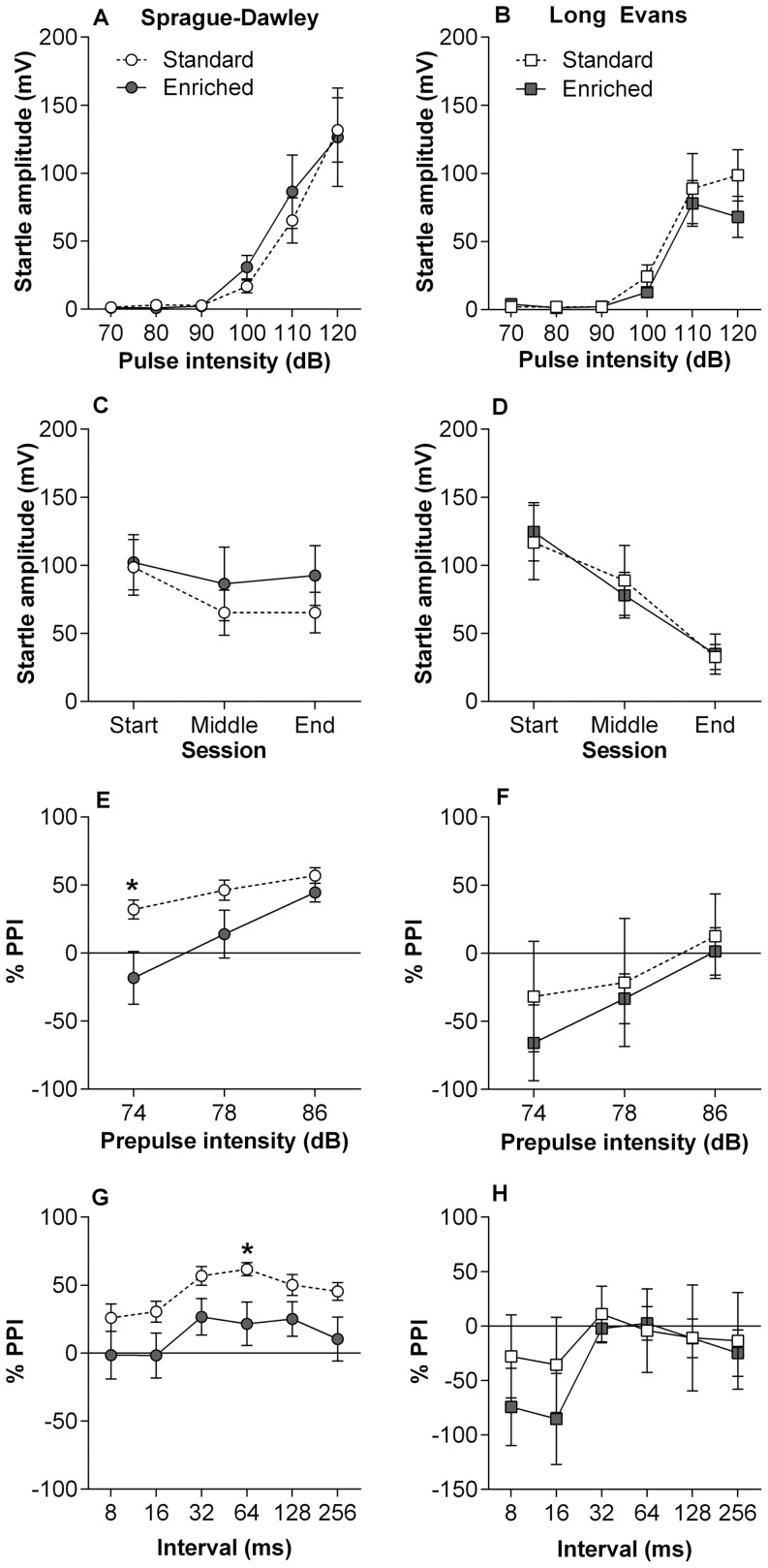
Pre-pulse Inhibition. (A,B) Acoustic startle response in SD (left panels) and LE (right panels) rats demonstrating increasing startle amplitude with louder acoustic pulses. (C,D) Habituation of the startle response to 110 db pulses presented at the start, middle and end of the session showing a clear within-session reduction in LE rats compared to SD rats, such that there was a main effect of strain at the end of the session (*F*_(1,30)_ = 7.96, *p* = .009). (E,F) %PPI is presented as each intensity (74, 78 and 86 dB) averaged across different six pre-pulse intervals. %PPI at 74 dB was significantly reduced in SD rats housed in enrichment compared to standard housed rats (*t*_(13)_ = −2.31, *p* = .038). Variability of PPI within LE rats was noticeably greater than in SD rats and no significant effect of housing was found. (G,H) %PPI for each pre-pulse interval (averages across the three intensities) shows reduced PPI at 64 ms in SD rats from enriched cages compared to standard housed SD rats (*t*_(8.27)_ = −2.40, *p* = .042) while LE rats from both housing conditions did not differ. Standard housing (open), enriched housing (closed). Data presented as mean ± S.E.M.*p<0.05.

All groups showed an increase in startle amplitude with increased pulse intensity, however using a repeated measures ANOVA no effect of Strain (*F*_(1,27)_ = 1.00, *p* = .326) or Housing (*F*_(1,27)_ = 0.08, *p* = .774) was detected (Fig.2a,b). Habituation was measured by comparing startle amplitude at the start and end of the session and while there was no difference between Strain or Housing at the start, at the end there was a main effect of Strain (*F*_(1,30)_ = 7.96, *p* = .009) but not Housing (*F*_(1,30)_ = 0.84, *p* = .368; Fig. 2c,d). % PPI was pooled across pre-pulse intervals for each of the three intensities and analysed using a repeated measures ANOVA. A main effect of Strain (*F*_(1,30)_ = 4.68, *p* = .040), but not Housing (*F*_(1,30)_ = 1.10, *p* = .304) was found (Fig.2e,f). Within strains it was found that enriched SD rats when compared to standard housed SD rats had impaired PPI at 74 dB (*t*_(14)_ = −2.31, *p* = .038). When pre-pulse interval was pooled across intensity there was also a reduction in PPI at 64 ms in SD from enriched cages compared to those from standard housing (*t*_(8.27)_ = −2.40, *p* = .042; Fig.2g,h).

### Operant training

**Figure 3:**
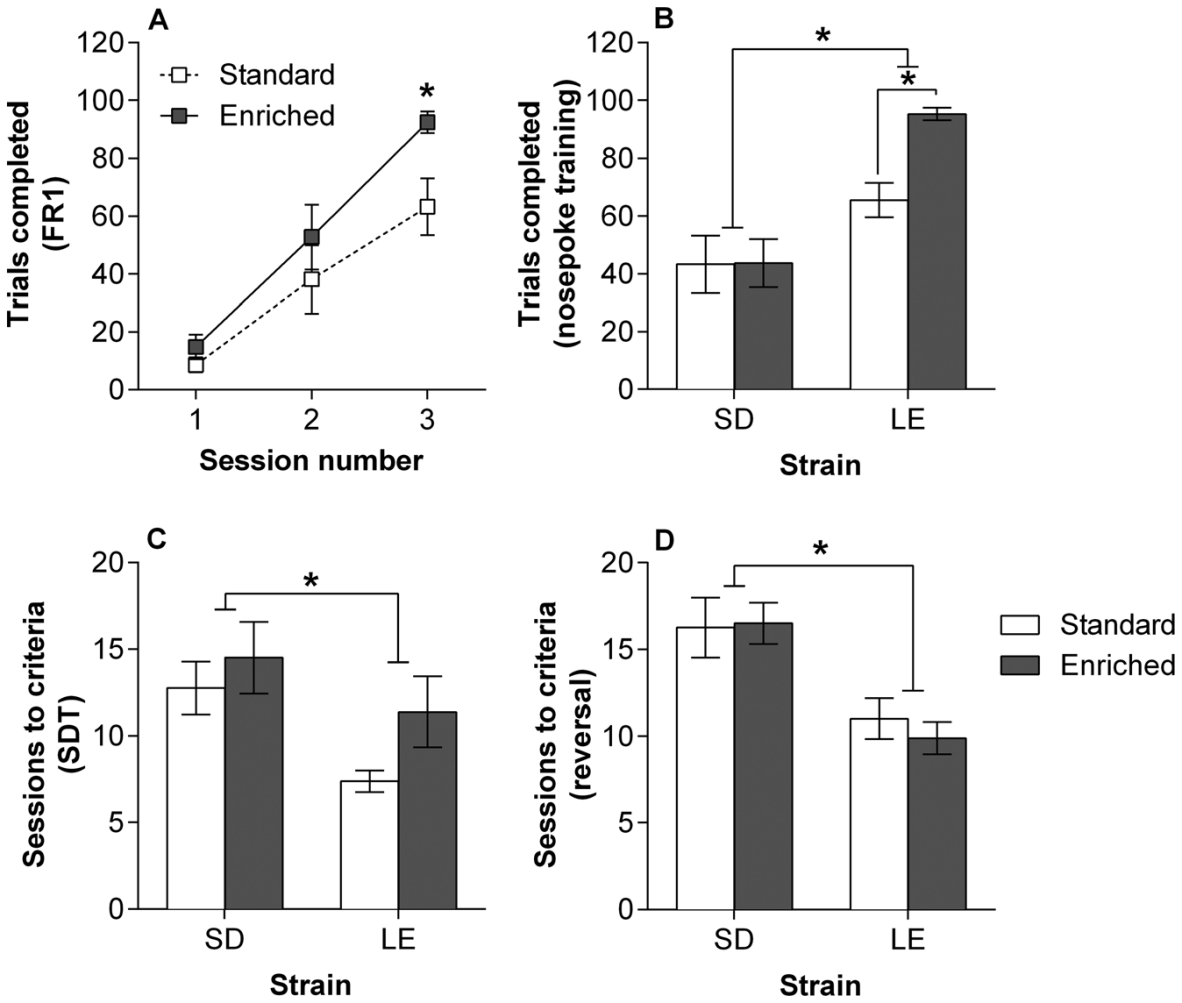
Operant training. (A) Fixed ratio (FR1) training in LE rats, showing the number of trials completed was greater in enriched rats compared to those from standard housing conditions after 3 days of training (*t_(13)_* = 2.41, *p* = .032). (B) On the first session of learning to nose poke to receive a food reward, LE rats completed more trials than SD rats (*F*_(1,31)_ = 26.3, *p*<0.001). Additionally, LE rats from enriched housing successfully performed more trials compared to those from standard housing (t(13) = 2.41, p = .032). (C) The number of sessions required to reach criteria on a signal detection task (SDT) was greater in SD rats compared to LE (F(1,31) = 6.47; p = .017). (D) LE rats were able to acquire the reversed contingency on SDT in fewer sessions than SD rats (F(1,31) = 21.21, p<0.001). Standard housing (open), enriched housing (closed). Data presented as mean ± S.E.M.*p<0.05.

After 3 days learning the initial protocol, where rats were required to respond with head entries to receive rewards, there was a significant interaction between Strain and Housing on the number of trials completed (F(1,31) = 5.59, p = .025); however there was no significant main effect of Strain or Housing. LE rats raised in enriched housing performed significantly more trials that those from standard housing conditions (t(13) = 2.41, p = .032; Fig.3a). Next rats learnt to nose poke a central aperture to receive a reward. On the first day of training there was a main effect of Strain (F(1,31) = 26.3, p<0.001) and Housing (F(1,31) = 4.39, p = .045), and a Strain*Housing interaction (F(1,31) = 4.18, p = .050). These results indicated that LE rats performed more successful trials than SD rats and that those from enriched housing performed better, which was most pronounced in LE rats (t(13) = 2.41, p = .032; Fig.3b). There was a main effect of Strain on the number of sessions required to learn the signal detection task (F(1,31) = 6.47, p = .017; Fig.3c) and the reversal (F(1,31) = 21.21, p<0.001; Fig.3d), finding in both cases that LE rats required less training than SD rats.

## Discussion

The genetic and environmental background of rodent models are known to influence behavioural phenotypes, however many animal models of schizophrenia use the same strain and standard housing conditions. In the current study we used two outbred rat strains, one used for modelling neuropsychiatric disorders (SD) and a strain more frequently used for cognitive testing (LE) and compared their behaviour when raised in standard or enriched cages. We found that strain and housing conditions influenced behavioural measures across a number of behavioural domains relevant to animal models of neuropsychiatric disorders; including altered locomotion, increased anxiety, impaired PPI and altered learning [39]–[42]. Importantly, these genetic and environmental manipulations may help us to understand complex phenotypes relevant to human neuropsychiatric disorders.

### Strain

Overall LE rats were more active, had greater exploration, reduced anxiety, reduced PPI and improved cognitive performance compared to SD rats. Sensorimotor gating was measured using PPI and LE rats showed greater habituated to the startle pulse than SD rats, however the increased variation observed in LE rats made PPI comparisons difficult. LE rats generally showed facilitation, whereas SD rats showed inhibition of the startle reflex. Facilitation can occur if the pre-pulse interval is too short or too long resulting in summation of the startle response. However, the same protocol has been used previously by our group using SD rats from a different supplier with similar results to the SD rats tested in this study [43]. Strain and supplier differences in PPI have been identified by a number of studies (e.g. [18], [20], [44]) however the facilitation observed in LE rats is likely to be a characteristic of the strain under these testing conditions.

When compared using an operant task it was found that SD rats required more training sessions than LE rats to learn to self initiate trials, to learn a signal detection task and to acquire reversal of task contingencies. Strain differences in the acquisition of operant behaviours have been observed previously in studies comparing lever press acquisition in LE, SD and Wistar strains [45]. All strains acquired the task, however albino strains took longer than LE rats to learn this behaviour. It is interesting to note the widespread use of pigmented strains for cognitive tasks, while most animal models of schizophrenia are developed in albino strains. Comparing strains that differ in cognitive performance within existing animal model preparations may provide a useful tool for modelling and understanding cognitive deficits relevant to schizophrenia.

### Housing

While some effects were strain-specific, it was found that minimal enrichment lead to a phenotype characterised by reduced anxiety-related behaviour, locomotion and PPI, and enriched rats completed more trials successfully during operant training. On the EPM enriched rats of both strains spent less time on the open arms than those from standard housing, which is indicative of greater anxiety. This is contrary to many studies showing increased open arm usage in rats from environmentally enriched cages [46], however reduced open arm time after enrichment has been seen in mice [47] and after access to wheel running in rats [48]. Latency to emergence on the light dark test was also used to measure anxiety and SD rats raised in the enriched environment emerged earlier than those raised in standard housing, a result found by others using Wistar rats and indicating enriched rats were less anxious [49]. Additionally, SD rats from enriched cages performed head dips on the hole board test earlier than those from standard housing. Previously, enrichment in SD rats has been associated with greater exploration and reduced corticosterone levels on the hole board test [46]. Together these results indicate enriched housing results in a less anxious phenotype. While this initially appears to conflict with the results from the EPM, the inclusion of a shelter in the enriched cages may have led to differential use of the protective zones.

Further analysis of ethologically relevant behaviours revealed that on the EPM, stretch-attend behaviour from the protected zone was performed earlier and more frequently in enriched rats. The stretch-attend posture is adopted when a rodent explores a novel or potentially dangerous space [50], indicating enriched rats used a protective posture to investigate the open arm rather than walking out. Rats from enriched housing also began grooming earlier and for longer than standard housed rats. Furthermore, although the duration of grooming was longer, the number of bouts was not the same, indicating that enriched rats were performing longer uninterrupted bouts of grooming. Increased grooming can be triggered in both high and low stress situations, however broken or rapid bouts of grooming are associated with increased stress in rats and mice [51]. These results suggest that rats from enriched housing are less anxious than standard housed rats. Reduced latency to head dip on the hole board and earlier emergence on the light dark test provide further evidence for reduced anxiety-related behaviours in rats from enriched cages.

Enriched LE rats had reduced locomotion on the open field test, which is in agreement with a previous study in mice comparing standard and enriched conditions using either a freely spinning or locked wheel. This study isolated reduced locomotion in the open field test to enriched caging with the addition of a functional running wheel [47]. The inclusion of enrichment toys, but without a running wheel, has also been associated with faster habituation and reduced locomotion in the open field test in SD rats when compared to socially housed controls [52].

Enrichment within SD rats was associated with a PPI deficit at the lowest pre-pulse intensity and this pattern was also observed at higher intensities and when split for pre-pulse interval. Some studies report no effect of enrichment [52], however other studies have reported reduced PPI at low pre-pulse intensities after environmental enrichment [46], and this deficit appears to be modulated by exercise in mice [47]. PPI is one of the most widely used tests for validating animal models of schizophrenia due to the translatability of this behaviour across many species and the well established deficit found in patients [53]. It was not expected that the simple environmental changes used in this study would disrupt PPI, but the results highlight the sensitivity of this test to environmental manipulations.

The acquisition of simple operant protocols was faster in LE rats raised in enriched cages compared to those from standard housing conditions. A study investigating performance on the Morris water maze found that enriched rats outperformed those from standard housing [49]. However, this result could be explained by the difference in thigmotaxis, indicating enriched rats were less anxious rather than having improved cognition. This is an important consideration, as changes in stress or anxiety will interfere with performance measures on behavioural tasks. Rats in the current study had been handled extensively prior to operant training, however greater anxiety may have contributed to the impaired learning observed in standard housed LE rats.

Environmental enrichment altered the behavioural phenotype of both strains, confirming that including a running wheel and shelter were sufficient to alter adult behaviour. This design does not result in the large changes in complexity or novelty used in other EE studies, but has increased the opportunity for voluntary exercise. While Sprague Dawley rats grew significantly larger than Long Evans rats, voluntary running did not alter body weight. This study used an enrichment protocol that was readily reproducible and required minimal equipment or labour. This makes improved housing more appealing, less expensive and reduces the variable experiences that would occur in a more dynamic environment. Investigating the compounding effects of GxE manipulations has become increasingly important for understanding neuropsychiatric conditions [54], [55]. Changing the background strain or housing environment of established animal models of schizophrenia may reveal important changes in behavioural outcomes and a better understanding of how GxE interactions impact on altered phenotypes [56].

While the order of testing has been shown to alter some behavioural outcomes [57], the consistency of the results across tests suggests the differences observed can be reliably assessed using a behavioural test battery. As testing on one paradigm can influence performance on later tests, the order of testing was determined based on the requirement for novelty-based responding and to reduce the effects of stress. Running a battery in a different order may change the outcomes, however a key benefit of using test batteries is that responses to a number of different challenges can be compared and the most robust phenotypic features can be determined. Three tests of cognition were used in this study with no difference in performance on the Y-maze, the RAM found overall effects of strain, and the operant tasks found the same main effect of strain but also that LE rats with enrichment acquired tasks faster. These results suggest that LE rats can outperform SD rats on various measures of cognition, but also that cognitive performance in LE was sensitive to environmental manipulations. With an increasing interest in cognitive phenotypes, strains that not only display deficits but that are also capable of enhancement should be used. The lack of strain variation used in rat models of neuropsychiatric modelling may need to be revised to answer questions about cognitive functioning and for the development of pro-cognitive treatments.

By comparing two strains commonly used for either neuropsychiatric animal models or cognitive studies, this study has highlighted that while SD rats have a less variable PPI response, they were outperformed by LE rats on cognitive tasks. Furthermore, the effects of enrichment on cognition were only apparent in LE rats. Using a strain that acquires cognitive tasks faster, makes fewer errors and shows greater sensitivity to environmental manipulations on cognitive measures may be beneficial when cognitive phenotypes are being explored.

Future studies should investigate whether changing strain or housing conditions alters the outcomes derived from manipulations relevant to some of the well-established animal models of neuropsychiatric disorders. Given the importance of understanding the GxE interaction in disorders, such as schizophrenia, it seems imperative that further consideration is given to the restricted range of strain and housing conditions being tested. Altering strain and housing conditions may provide important clues to help us understand how GxE interactions ultimately lead to changes in behavioural phenotypes relevant to human disorders.

## Supporting Information

Checklist S1 **PRISMA checklist.**
(DOC)
